# Internal Use of Chloroform

**Published:** 1854-03

**Authors:** Henry Hartshorne

**Affiliations:** Philadelphia


### *On the internal use of Chloroform.*

BY HENRY HHRTSHORNE, M. D., PHILADELPHIA.

Since 1848, when some account was given in this journal of experiments with chloroform, internally administered, it has been variously and extensively used by practitioners in different parts of the world. It is now generally recognised as being, when so used, a narcotic of the mildest and yet most powerful character, and as possessing in its pungency, also, a quality which recommends it in some cases above other anodynes.

The object of this article is chiefly to make some remarks upon its dose and mode of administration. Many practitioners within the writer's knowledge hesitate, from their recollection of its power as an anæsthetic to give it in doses of more than a few drops; and as the drop is exceedingly small, such doses are really often insignificant.—The writer can assert, from positive experience, that a fluidrachm of chloroform, taken by the stomach, is not more than equal, in soporific effects, to 30 or 35 drops of laudanum. In doses of 50 to 75 drops (about 15 minims), I have given it every half hour for several hours together. It differs from the opiate preparations in the promptness of its hypnotic action, the much shorter period of its duration, a less degree of cerebral oppression, and the absence of all stimulus to the circulation. It might be called a 'diffusible narcotic,' comparing in this respect with opium as ammonia does with alcohol. To produce much effect with it, repeated doses, at short intervals, will be necessary.

The pungent property, already alluded to, causes it to require plentiful dilution, which is, of course, facilitated by the addition of some demulcent. Perhaps the orgeat syrup is the best. Every fluidrachm of chloroform should have at least two fluid ounces of water with it when taken; and it will need, if in ordinary gum mucilage, considerable agitation to resuspend the particles immediately before swallowing. When taken in aqueous mixture alone, however, unless in very small doses, it produces nausea with some persons. This is entirely prevented by the addition of a strong aromatic, or, still better, by giving the chloroform in aromatic tincture. From the ready solution and kindred action of camphor with chloroform, their combination has become a very common one. For many purposes, however, a still better preparation is a sort of chloroform paregoric, or tincture of chloroform, *e. g.*; ℞ Chloroform f℥ij; sp. camph. et tint. opii. aa f℥iss; Ol. cinnamom. gtt. viij. alcohol f℥iij. M. et fiat tinctura. Dose from 5 to 30 minims, or more, as required.

The most admirable effects have been witnessed from the administration of chloroform, as above combined, in malignant cholera. In the summer of 1849, my attention was first called to it while attending a very severe case of cholera with the late Prof. W. E. Horner. The prompt and signal restoration accomplished in that case, from a state of collapse, was evidently due to the exhibition by Prof. Horner, every five minutes, of a few drops of a combination of chloroform, oil of camphor, and laudanum, with ice, and warm frictions, externally. The writer's conviction was very strong that the short interval between the doses was an important item in the treatment.—*Am. Jour. Med. Sciences.*

